# Weak Knock Characteristic Extraction of a Two-Stroke Spark Ignition UAV Engine Burning RP-3 Kerosene Fuel Based on Intrinsic Modal Functions Energy Method

**DOI:** 10.3390/s20041148

**Published:** 2020-02-19

**Authors:** Jing Sheng, Rui Liu, Guoman Liu

**Affiliations:** 1 Jiangxi Province Key Laboratory of Precision Drive & Control, Nanchang Institute of Technology, Nanchang 330099, China; jerry@nit.edu.cn; 2 School of Mechanical and Power Engineering, Nanjing Tech University, 211816 Nanjing, China; timliu@njtech.edu.cn

**Keywords:** intrinsic modal functions energy (IMFE) method, a two-stroke spark ignition UAV engine, engine block vibration signals, weak knock characteristic extraction, wavelet packet energy method

## Abstract

To solve the problem of the weak knock characteristic extraction for a port-injected two-stoke spark ignition (SI) unmanned aerial vehicle (UAV) engine burning aviation kerosene fuel, which is also known as the Rocket Propellant 3 (RP-3), the Intrinsic modal Functions Energy (IMFE) method is proposed according to the orthogonality of the intrinsic modal functions (IMFs). In this method, engine block vibration signals of the two-stroke SI UAV engine are decomposed into a finite number of intrinsic modal function (IMF) components. Then, the energy weight value of each IMF component is calculated, and the IMF component with the largest energy weight value is selected as the dominant characteristic component. The knock characteristic frequency of the two-stroke SI UAV engine is obtained by analyzing the frequency spectrum of the dominant characteristic component. A simulation experiment is designed and the feasibility of the algorithm is verified. The engine block vibration signals of the two-stroke SI UAV engine at 5100 rpm and 5200 rpm were extracted by this method. The results showed that the knock characteristic frequencies of engine block vibration signals at 5100 rpm and 5200 rpm were 3.320 kHz and 3.125 kHz, respectively. The Wavelet Packet Energy method was used to extract the characteristics of the same engine block vibration signal at 5200 rpm, and the same result as the IMFE method is obtained, which verifies the effectiveness of the IMFE method.

## 1. Introduction

Two-stroke piston engines are widely used in UAV and light aircraft due to their simple structure, small size, and good fuel economy [1]. Most aviation devices prefer SI engines because the compression ignition (CI) engine is heavier and the weight-to-weight ratio is not as good as that of an SI engine [2]. In general, two-stroke UAV SI piston engines use aviation gasoline as fuel because aviation gasoline has a sufficiently low crystallizing point, good evaporation, and knock resistance. However, aviation gasoline is characterized by a low flash point and high volatility, and it is prone to explosion when exposed to open fire at room temperature, which poses a great safety hazard for fuel storage, transportation, and use [3]. Especially in certain areas with high requirements on fuel safety, it is urgent to find safe and reliable fuel to replace aviation gasoline fuel.

As an alternative heavy fuel to SI engines, aviation kerosene has similar physical and chemical properties [4]. Compared with aviation gasoline, aviation kerosene fuel, that is, Rocket Propellant 3 (RP-3), has a high flash point, weak volatility, good thermal stability, high energy density, and good safety performance [5,6]. In recent years, SI engines capable of using aviation kerosene (RP-3) have been studied and widely used in the military in order to satisfy the ‘single-fuel’ policy proposed by the Department of the Defense. At present, RP-3 kerosene fuel is the most widely used fuel for military use in China. Some properties of aviation gasoline and RP-3 are compared in Table 1 [7,8].

As shown in Table 1, the octane number and auto-ignition temperature of RP-3 are both lower than those of aviation gasoline, which means when the SI engine uses RP-3 as its fuel, it is more prone to knock. Therefore, knock has become a major obstacle for the development of SI engines when burning RP-3 fuel [9].

Knock is defined as abnormal combustion induced by auto-ignition in the combustion chamber. It is generally accepted that knock is caused by the auto-ignition of the end-gas [10]. In the combustion chamber of SI engines, the flame front propagates from the ignition source (around the spark plug) to the end-gas [11,12]. The temperature and pressure of the end-gas increase during the propagation of the flame, and if the temperature becomes too high, the end-gas may ignite spontaneous, which results in knock combustion [13,14,15]. When knock happens, the chemical energy stored in the end-gas will be released quickly and the disordered pressure waves will be generated rapidly, which will lead to following results [16]: (1) the thermal conductivity between the gas and the chamber wall will be increased, (2) the thermal load of the engine part will also be increased, (3) the thermal efficiency of the cycle will be reduced, and (4) the severe structural damage of the engine will occur.

In the study, the research team of the author found that the anti-knock performance of RP-3 kerosene fuel was poor [17,18]. Strong knock occurs easily when the two-stroke SI UAV engine burns RP-3 kerosene fuel. Strong knock will not only restrict the improvement of power and economy of the SI UAV engine, but even damage the engine body [19]. However, weak knock can increase the power output and reduce the fuel consumption [20]. Therefore, it is of great significance to detect and recognize the knock state of the two-stroke SI UAV engine burning RP-3 kerosene fuel in real time and extract the weak knock characteristics.

When the knock occurs, high frequency oscillation pressure waves will be created within the combustion chamber and induce high frequency vibration of the cylinder block [21]. The popular and valid approach is to measure knock impact by using several types of sensors such as pressure sensors and acceleration sensors. Cylinder pressure oscillations clearly indicate what happens during a knock cycle inside the combustion chamber. However, the cylinder pressure sensors are very expensive. Therefore, the most widely used method of measuring knock is using a simple acceleration senor attached to the cylinder block. This method is an easy and cost-effective task. However, vibrations induced by resonances in the combustion chamber have to be detected against a complex background of heavy noise and other vibrations. The vibration signal needs to be reprocessed.

Signal transform techniques are useful tools for knock characteristic extraction methods, such as Fast Fourier Transform (FFT [22]), Short-time Fourier Transform (STFT [23]), Wigner Ville Distribution (WVD [24]), Continuous Wavelet Transform (CWT [25,26]), and Discrete Wavelet Transform (DWT [27]) have been applied to analyze engine vibration signals for knock detection for the past few decades. By considering that knock in the engine makes impact-induced resonant vibrations of the cylinder head or engine block, the collected vibration signal essentially has non-stationary and nonlinear properties. Thus, direct use of the FFT is fast, and it offers the appropriate noise filtering capabilities, but it is not suitable for identifying knock characteristics with rapid changes in both the time and frequency components. Time-frequency analysis methods such as STFT, WVD, and CWT are capable of dealing with a non-stationary signal. However, the computational intensity of such time-frequency representations in two-dimensional space inhibits their implementation for real-time applications. The DWT is a popular tool for time frequency analysis and focuses extensively on extracting transient characteristics from a complicated signal background and identifying machinery faults even in the initial stages. The DWT-based knock detection methods are also popular because of their fast algorithms. However, the selection of an optimum wavelet basis depends on the characteristics of the signal to be detected, which is difficult to generalize and potentially sensitive to background noise [28]. With classical DWT, fixed scale filters and wavelet filters are utilized to generate approximations and detail coefficients in each scale. Unfortunately, vibration signals collected from engines are non-stationary, and the structures vary significantly within each scale. Therefore, using a single wavelet filter cannot fit the local characteristics sophisticatedly. Accordingly, the ability to extract transient features of engine knock, especially in the early stages, is undermined.

In addition, how to find a suitable wavelet basis function as the knock characteristic generating function is also a difficult problem for wavelet transform. At present, the number of wavelet basis functions is infinite. Different wavelet basis functions are selected to process the same signal, and different results will be obtained. The proper selection and optimization of the wavelet basis function are worth discussing, but, so far, there is no systematic method to summarize. Thus, the selection of the wavelet basis function is influenced by human factors to a great extent. After selecting the wavelet basis function and a decomposition scale, the result of wavelet transform is a certain fixed frequency band signal. Therefore, the frequency range of wavelet transform cannot adaptively follow the change of the signal, which results in a clear signal redundancy. These problems of wavelet transform seriously affect the accuracy of weak knock characteristic extraction.

In order to accurately and effectively extract the weak knock characteristic frequency from engine block vibration signals, a new characteristic extraction method, namely the IMFE method, is adopted in this paper. The IMFE method is an adaptive nonlinear non-stationary signal time-frequency analysis method, which is a major breakthrough for linear and steady-state spectral analysis based on Fourier Transform. The IMFE method can decompose signals into the sum of several orders of IMFs, according to different scales or trends [29]. Each IMF component changes with the change of the signal and has strong adaptability. The energy weight values of each IMF component are calculated, and the IMF component with the largest energy weight value is selected as the dominant characteristic component, which is used to extract the important characteristic information contained in the signal by Fast Fourier Transform (FFT).

The IMFE method has been gradually applied in the fields of rolling bearing fault recognition [30], plunger pump fault diagnosis [31], and respiratory signal feature extraction [32]. It has achieved good results. The IMFE method does not need to set a fixed basis function, and each IMF component is determined by the signal characteristics. Therefore, it has good adaptability. Since the IMF component with the largest energy weight value contains the important characteristic information of the original signal, the characteristic frequency of the original signal can be obtained from the IMF component by FFT. The IMFE method is fast and easy to implement. Compared with wavelet transform, the IMFE method has higher precision and is suitable to detect the characteristic frequency of the knock signal in a lower signal-noise ratio. In this paper, the IMFE method is applied to extract the weak knock characteristics of the two-stroke SI UAV engine burning RP-3 kerosene fuel, which provides the theoretical basis for the realization of knock real-time control and the engine performance optimization.

## 2. Method

The IMFE method is based on an Empirical Mode Decomposition (EMD) method. The EMD method can decompose the non-stationary and non-linear signals into a set of steady and linear data series, namely IMFs. The IMFs must satisfy two conditions [33,34]: (1) for a column of data, the number of extreme points is equal to or at most different from the number of zeros, (2) at any point, the mean value of the envelope line composed of local maximum points and local minimum points is zero.

The EMD method uses the envelope of local maximum and minimum to obtain IMF components. Once all extremum points are obtained, all local maxima are interpolated using cubic spline interpolation to form an upper envelope of data [35]. Similarly, all local minima are interpolated to form the lower envelope of the data. To extract IMFs, an iterative process is described below [36,37].

The average value of the upper envelope and lower envelope is denoted as $m_{1}(t)$, and the original data s(t) minus $m_{1}(t)$ gives $h_{1}(t)$.

(1) $$s(t)-m_{1}(t)=h_{1}(t)$$

The process is repeated k times until $h_{1}(t)$ meets the IMF’s definition requirement and the average value is close to zero. Therefore, the first IMF component $c_{1}(t)$ can be obtained, which represents the highest frequency component of the signal s(t).

(2) $$h_{1(k-1)}(t)-m_{1k}(t)=h_{1k}(t)$$

(3) $$c_{1}(t)=h_{1k}(t)$$

$c_{1}(t)$ is separated from s(t). A difference signal $r_{1}(t)$ that removes the high-frequency component is obtained.

(4) $$r_{1}(t)=s(t)-c_{1}(t)$$

Using $r_{1}(t)$ as the original data, the above steps are repeated to obtain n IMF components $c_{n}(t)$.

(5) $$r_{n-1}(t)-c_{n}(t)=r_{n}(t)$$

Equation (6) can be obtained when $c_{n}(t)$ or $r_{n}(t)$ meets the given termination conditions (usually making $r_{n}(t)$ a monotone function).

(6) $$s(t)=\sum_{j=1}^{n}c_{j}(t)+r_{n}(t)$$

IMF components at different levels contain different frequency components. The lower the order of the IMF component, the more high-frequency components it contains. For the IMF component of the same order, different types of signals have different frequency components. Therefore, IMF component energy can be used as a feature for classification. The indicators of signal energy are defined as follows [38].

(7) $$E_{0}={\int_{t_{0}}^{t_{1}}\left|s(t)\right|}^{2}dt$$

(8) $$E_{n}=E_{r}+\sum_{i=1}^{n}E_{f}(i)$$

(9) $$\varepsilon =\left[(E_{n}-E_{0})/E_{0}\right]\times 100\%$$

(10) $$\eta (i)=\left\{E_{f}(i)/\left[\sum_{i=1}^{n}E_{f}(i)\right]\right\}\times 100\%$$

where $E_{0}$ is the original signal energy, $E_{n}$ is the sum of the energy of all IMF components obtained by the EMD method, $E_{r}$ is the energy of the remaining components, $E_{f}(i)$ is the i-order IMF component energy, n is the IMF component order, ε is the energy error evaluation index, and η(i) is the energy weight value of the i-order IMF component.

In the process of the IMFE method, if the energy error exceeds the allowable range, the boundary extension and fitting algorithm are modified. The decomposition is then redone, and the process is repeated until ε meets the requirements. This kind of closed-loop self-inspection can effectively restrain the false components produced in the process of decomposition, and provides a prerequisite for judging the dominant characteristic component according to the energy weight value η(i).

## 3. Simulation Example

To verify the effectiveness of the IMFE method in this paper to extract the knock characteristic frequency, a simulation calculation is set up. The knock simulation signal S(t) is composed of two parts, which include the simulation signal component k(t) and the noise component N.

(11) S(t)=k(t)+N

**Figure 1:**
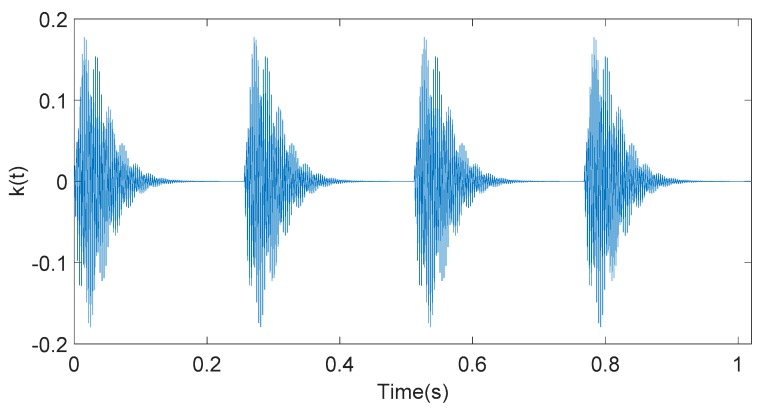
Time-domain waveform of the simulation component signal k(t).

The simulation signal component k(t) can be simulated by an exponentially decaying sinusoidal signal.

(12) $$k(t)=\left\{ \begin{array}{l} A(t-t_{0})e^{-b{\left(t-t_{0}\right)}^{2}}\sin(2\pi ft+\phi );t\geq t_{0}\\ 0;t<t_{0} \end{array}\right.$$

where $A(t-t_{0})e^{-b{\left(t-t_{0}\right)}^{2}}$ represents the amplitude attenuation component of the signal component k(t). The frequency f is 350 Hz. The initial phase is φ = 0, and other parameters are A = 50, b = 100, and $t_{0}$ = 0 s, respectively. The sampling frequency is 1000 Hz. The simulation signal component k(t) described by the above parameters is shown in Figure 1.

**Figure 2:**
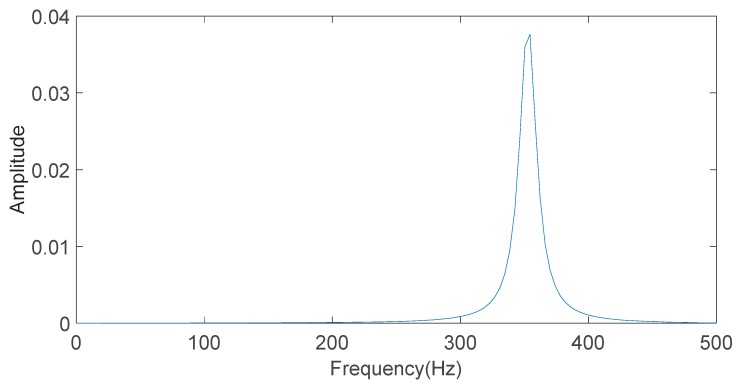
The frequency of the simulation component signal k(t) obtained by Fast Fourier Transform (FFT).

FFT is used to analyze the simulation signal component k(t) in order to calculate the frequency of the signal. The result is shown in Figure 2. It can be seen from Figure 2 that the frequency of the simulation signal component k(t) is 350 Hz, which is consistent with the parameters set.

**Figure 3:**
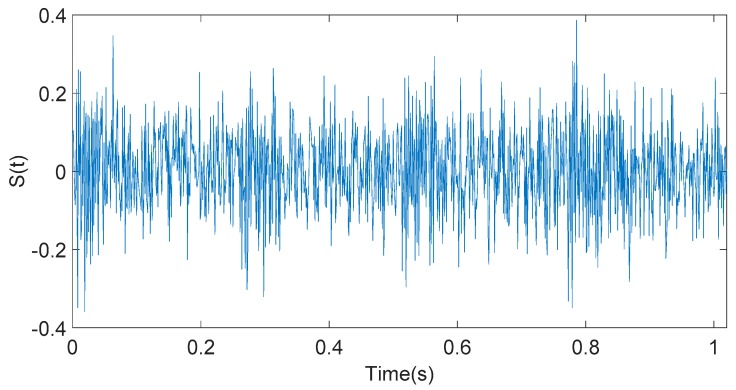
Time-domain waveform of simulation signal S(t).

The simulation signal component k(t) is mixed with the gaussian white noise component N to form the knock simulation signal S(t), as shown in Figure 3.

**Figure 4:**
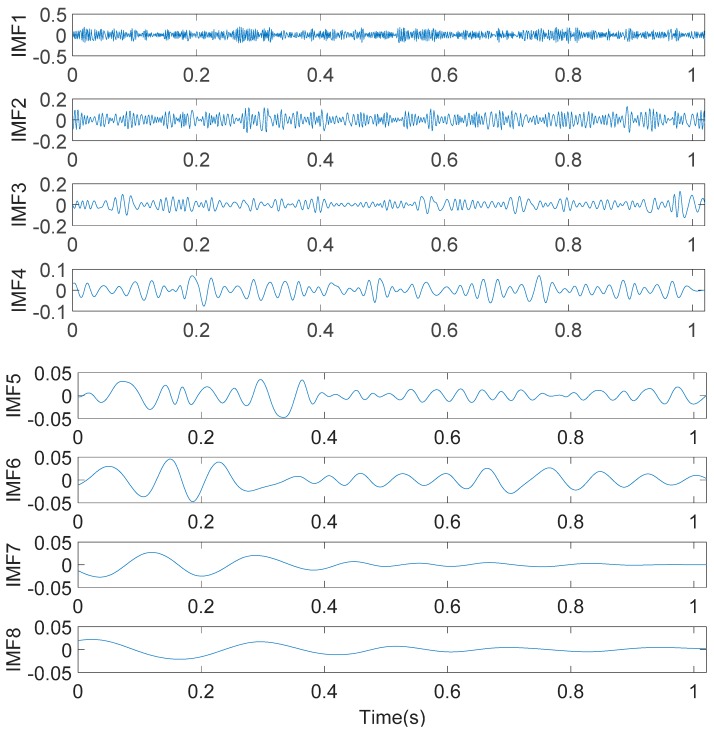
The time-domain diagrams of eight Intrinsic modal Function (IMF) components.

To analyze the time-frequency characteristic of the simulation signal S(t), the IMFE method is used for characteristic extraction. First, the simulation signal S(t) is decomposed. Thus, eight IMF components and one residual component are obtained. The time-domain diagrams of eight IMF components are shown in Figure 4.

**Figure 5:**
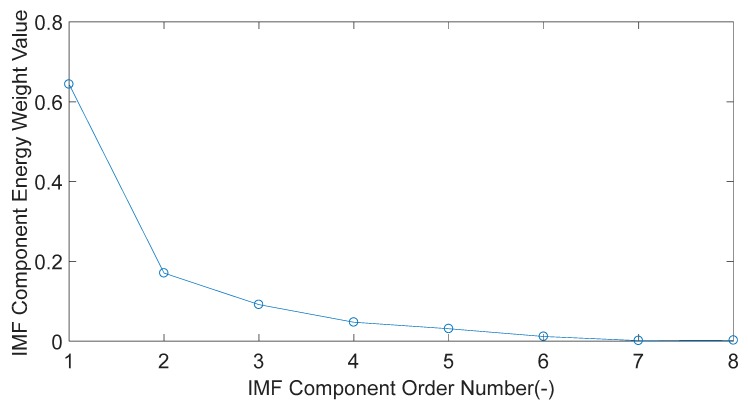
The energy weight value of eight Intrinsic modal Function (IMF) components.

In Figure 4, using the IMFE method, the simulation signal S(t) is decomposed according to different time scales. The high-frequency IMF component is decomposed first, and the low-frequency IMF component is decomposed in turn. A total of eight IMF components are obtained. The energy weight value of eight IMF components are calculated respectively, as shown in Figure 5.

**Figure 6:**
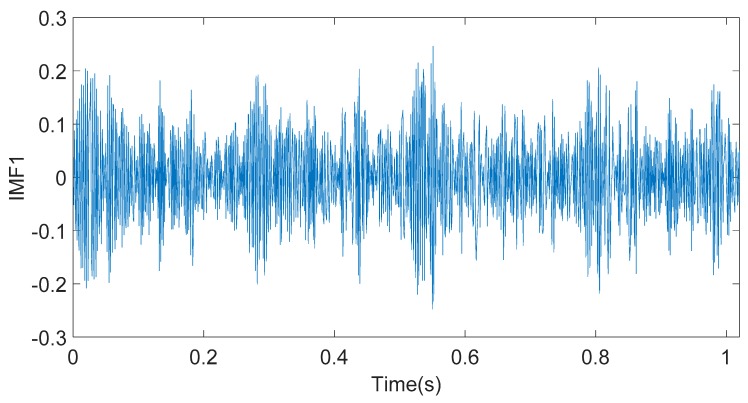
Time-domain waveform of the IMF1 component.

**Figure 7:**
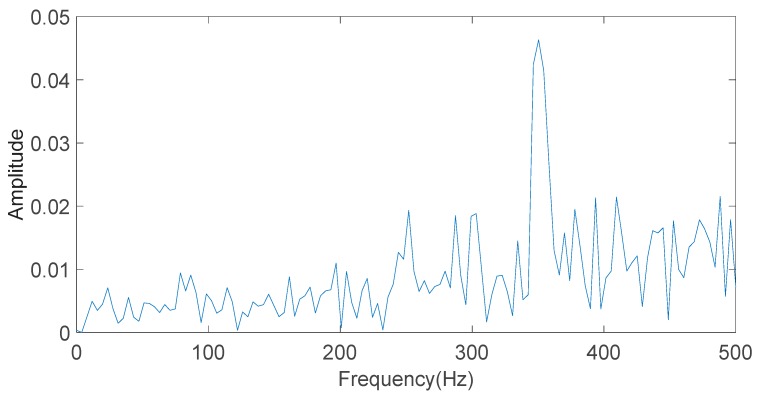
The frequency of the IMF1 component obtained by Fast Fourier Transform (FFT).

As shown in Figure 5, the energy of the simulation signal S(t) is mainly concentrated in the high-frequency component. The IMF1 component has the largest energy value, which is regarded as the dominant characteristic component. The time-domain diagram of the IMF1 component is shown in Figure 6. FFT is used to analyze the IMF1 component in order to calculate the frequency of the component. The result is shown in Figure 7. From Figure 7, it can be found that the signal part with a frequency of 350 Hz is mainly concentrated in the IMF1 component.

In conclusion, the IMFE method can be used to decompose the knock simulation signal into a series of IMF components adaptively in the frequency domain. According to the different energy proportion of each IMF component, the IMF component with the largest energy proportion is selected as the dominant characteristic component. The characteristic frequency value of the knock simulation signal can be obtained by FFT. The validity and feasibility of the IMFE method to extract the characteristics of knock simulation signals are verified.

## 4. Experiment Setup

### 4.1. Test Engine

A two-cylinder two-stroke spark ignition piston engine burning RP-3 kerosene fuel was used to conduct the experiments in this study. Table 2 shows the test engine specifications. °CA ADTC refers to crank angle after top dead center.

### 4.2. Test Bench

**Figure 8:**
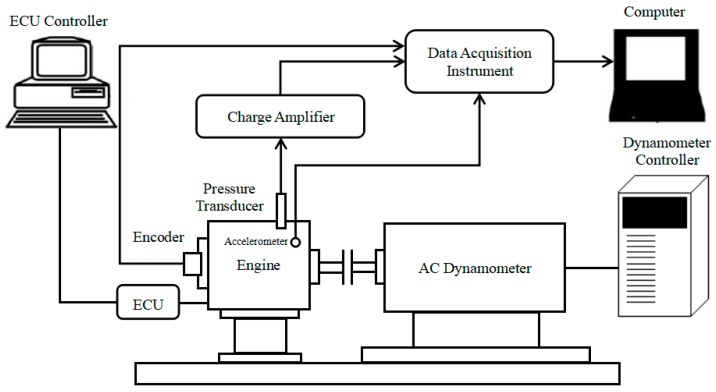
Schematic of the engine test bench.

**Figure 9:**
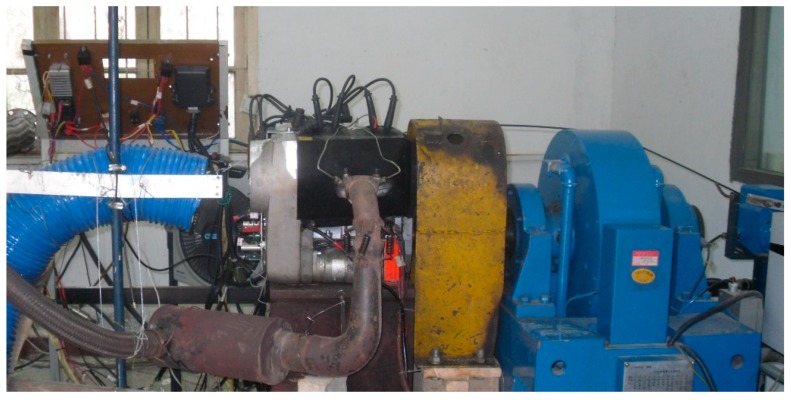
Image of the engine test bench.

Figure 8 shows the engine test bench layout and Figure 9 shows the image of the test bench. A DH151 piezoelectric acceleration sensor is mounted on the engine block of the two-stroke SI UAV engine to collect engine block vibration signals. The cylinder pressure sensor selected is Kistler6125A type spark plug pressure sensor. A DH5902 high-speed data acquisition instrument is adopted for the acquisition of engine block vibration signals and cylinder pressure signals.

## 5. Analysis of Test Results

**Figure 10:**
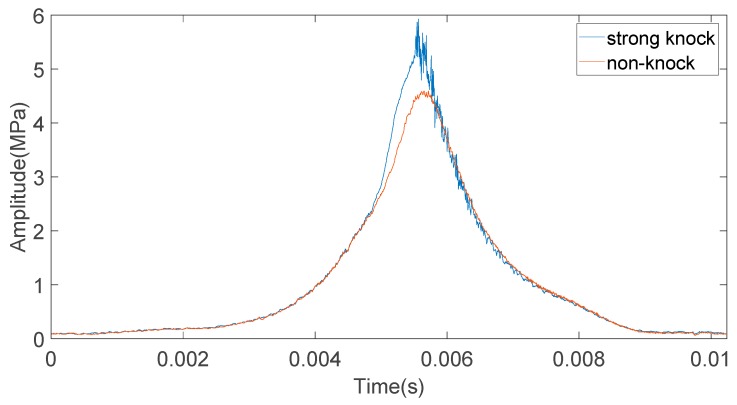
Curve of the cylinder pressure signal with strong knock and non-knock.

One of the important characteristics of knock is the high frequency oscillation of cylinder pressure signals. When knock occurs, the pressure and temperature in the cylinder suddenly increase. The pressure shock wave repeatedly impacts the cylinder wall, the top of the piston, and the cylinder head at an extremely high rate, which causes high-frequency pressure oscillation in the cylinder. For certain spark ignition engines, the frequency of high frequency pressure oscillation caused by knock is within a certain range. The amplitude of high frequency pressure oscillation after the peak of the pressure signal in the cylinder is related to the strength of the knock. Figure 10 shows the cylinder pressure signals obtained by a cylinder pressure sensor when the engine has non-knock and strong knock. As shown in Figure 10, when strong knock occurs, the peak value of the pressure signal in the cylinder increases significantly, which is accompanied by a high-frequency pressure signal oscillation.

During the test, the inlet temperature is 301 K and the engine operating condition are 5100 rpm and 5200 rpm. In order to avoid spectrum aliasing, the sampling frequency of the signal is selected as 200 kHz in order to maintain the high-frequency characteristics of the signals. The DH5902 high-speed data acquisition instrument is adopted to synchronously collect cylinder pressure signals and engine block vibration signals. The operational conditions of the test are shown in Table 3. A self-developed electronic control unit (ECU) was applied to enable free control over the engine operation parameters, such as the injection pulse width, injection timing angle, and ignition timing angle. Knock of the engine is gradually induced by increasing the ignition timing angle.

### 5.1. Knock Characteristic Extraction at 5100 rpm

**Figure 11:**
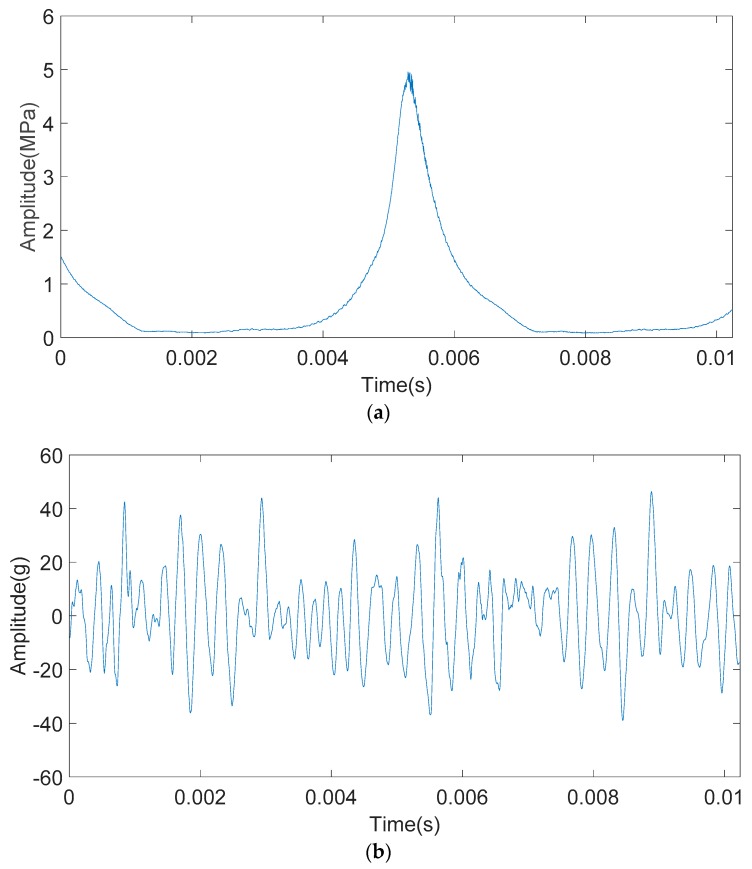
Time-domain waveform of the cylinder pressure signal and the engine block vibration signal under a weak knock condition at 5100 rpm. (**a**) Time-domain waveform of the cylinder pressure signal under a weak knock condition at 5100 rpm. (**b**) Time-domain waveform of the engine block vibration signal under weak knock condition at 5100 rpm.

Figure 11 shows the cylinder pressure and the engine block vibration signal under knock condition of the two-stroke SI UAV engine. From Figure 11a, it can be seen that the level of the high-frequency pressure oscillation is between non-knock and strong knock. As the cylinder pressure signal and the engine block vibration signal in Figure 11 are acquired synchronously, it is known that the cylinder pressure signal and the engine block vibration signal in Figure 11 are signals collected when weak knock occurs.

**Figure 12:**
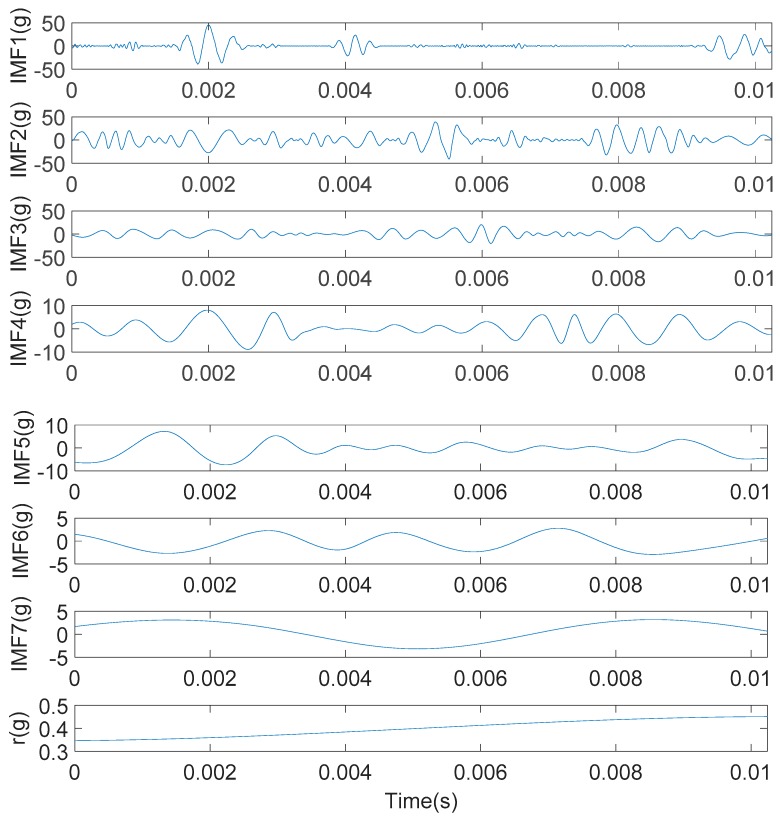
The intrinsic modal function (IMF) component of the engine block vibration signal under a weak knock condition at 5100 rpm.

The engine block vibration signal under weak knock condition shown in Figure 11b were decomposed by the IMFE method, and the results were shown in Figure 12. IMF1-IMF7 is the IMF component and r is a residual component.

Since the IMFE method does not require setting the base function, the extraction of IMF components in each order is determined by the signal characteristics, so it is adaptive. From Figure 12, IMF components obtained by the IMFE method gradually decrease in central frequency with the increase in order. Each IMF component contains different time feature scales and can display signal features with different resolutions, which indicates that the resolution in the IMFE method is adaptive.

In addition, the Index of Orthogonal (IO) of IMF components obtained by the IMFE method can be defined by the equation below [39].

(13) $$IO=\sum_{t=0}^{T}\left[\sum_{i=1}^{n+1}\sum_{j=1}^{n+1}c_{i}(t)c_{j}(t)/x^{2}(t)\right]$$

where T is the total signal length, and i≠j.

The orthogonality of IMF components obtained by the IMFE method was tested, and the IO value was 0.0921% (<1.0%), which indicates that the IMFE method was basically orthogonal. Based on this, it can be said that the energy of the engine block vibration signal under a weak knock condition is basically conserved in the process of decomposition, and the energy leakage between IMF components obtained by decomposition is very weak.

**Figure 13:**
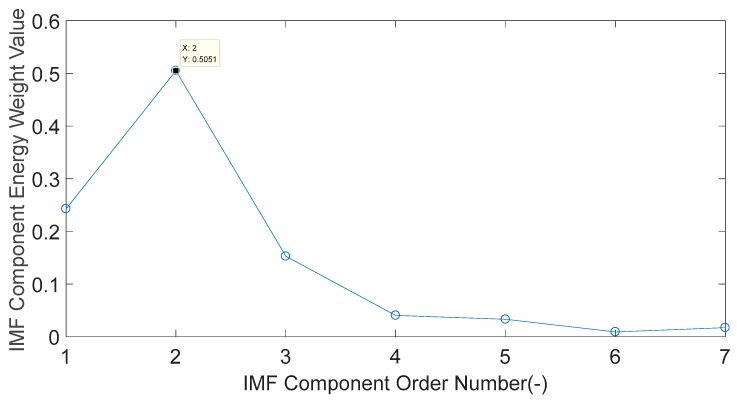
Each IMF component energy weight value of the engine block vibration signal under a weak knock condition at 5100 rpm.

The energy weight value of IMF components in order 1–7 is calculated, and the results are shown in Figure 13. As shown in Figure 13, the energy weight value of the IMF2 component accounts for 50.51% of the total energy, which is the dominant characteristic component.

**Figure 14:**
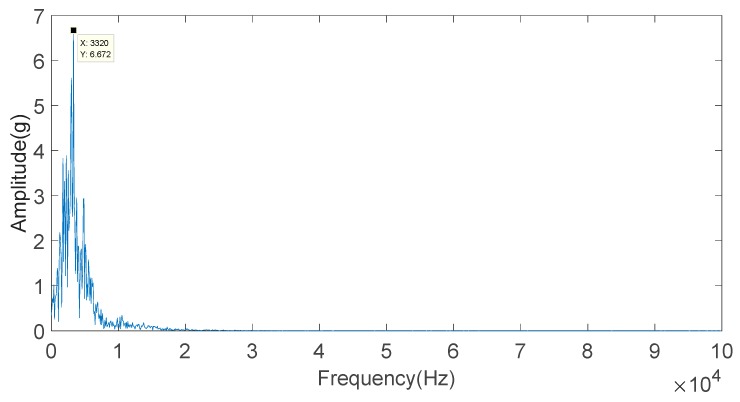
Frequency-domain diagram of the IMF2 component of the engine block vibration signal under a weak knock condition at 5100 rpm.

FFT is used to analyze the IMF2 component in order to calculate the frequency of the component, and the result is shown in Figure 14. Looking at Figure 14, the knock characteristic frequency of the engine block vibration signal under a weak knock condition at 5100 rpm is 3.320 kHz.

### 5.2. Knock Characteristic Extraction at 5200 rpm

**Figure 15:**
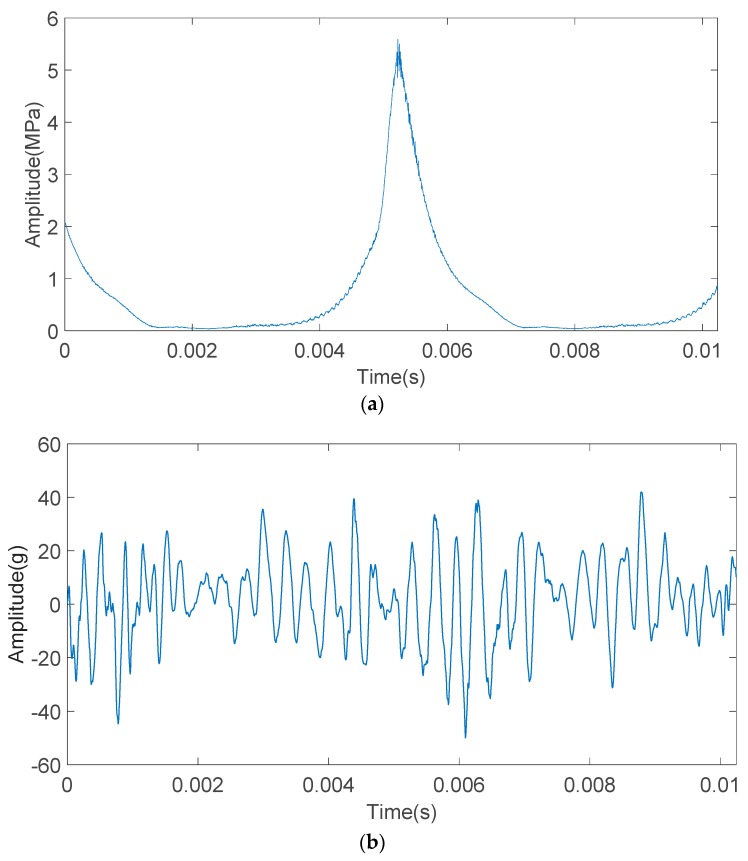
Time-domain waveform of the cylinder pressure signal and the engine block vibration signal under a weak knock condition at 5200 rpm. (**a**) Time-domain waveform of the cylinder pressure signal under a weak knock condition at 5200 rpm. (**b**) Time-domain waveform of the engine block vibration signal under a weak knock condition at 5200 rpm.

The time-domain diagram of the cylinder pressure signal and the engine block vibration signal under a weak knock condition collected synchronously at 5200 rpm is shown in Figure 15. Figure 15a is the time domain diagram of the cylinder pressure signal. Figure 15b shows the time domain diagram of engine block vibration signals. It can be seen from Figure 15 that the amplitude of the cylinder pressure signal and the engine block vibration signal under a weak knock condition at 5200 rpm are both higher than that at 5100 rpm.

**Figure 16:**
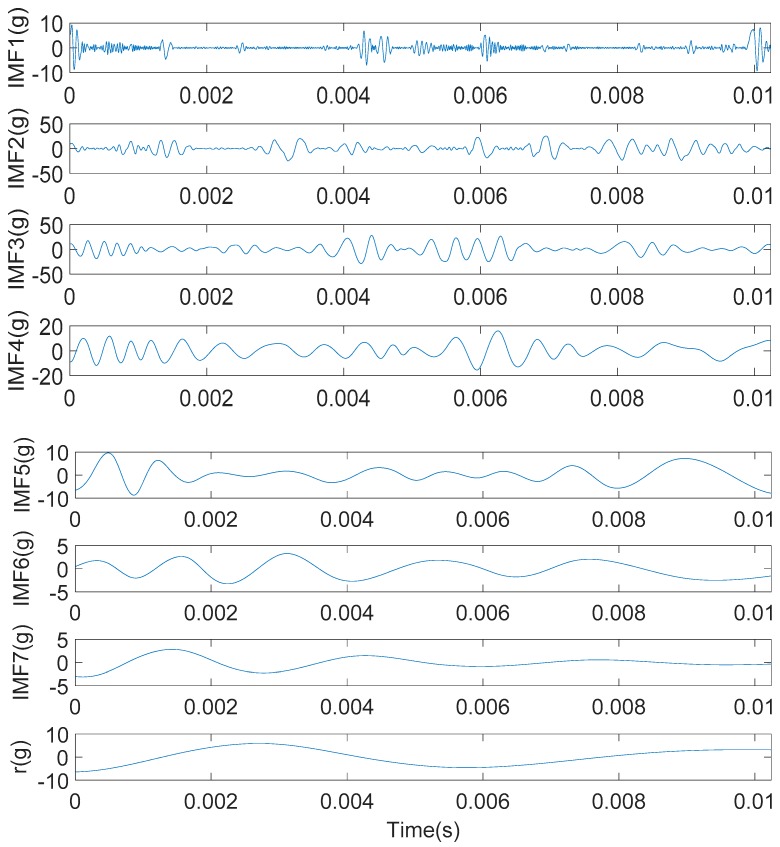
IMF component of the engine block vibration signal under a weak knock condition at 5200 rpm.

Using the IMFE method, the engine block vibration signal in Figure 15b is decomposed, and seven IMF components and one residual component are obtained, as shown in Figure 16. The orthogonality test of the IMF component is conducted, and the IO value is 0.1456% (<1.0%).

**Figure 17:**
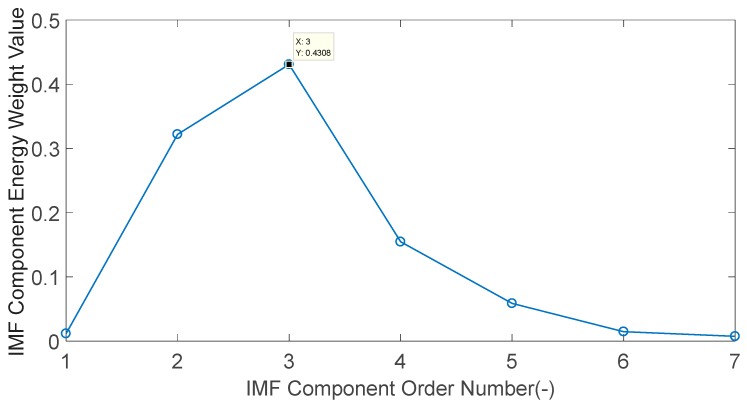
Each IMF component energy weight value of the engine block vibration signal under a weak knock condition at 5200 rpm.

The energy weight value of seven IMF components shown in Figure 16 are calculated, and the results are shown in Figure 17. Among them, the component with the maximum energy value is IMF3, which has an energy value of 43.08%. IMF3 is the dominant characteristic component.

**Figure 18:**
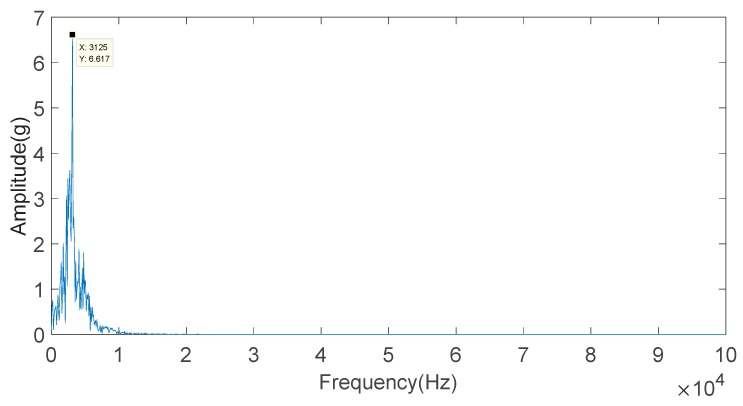
Frequency-domain diagram of the IMF2 component of the engine block vibration signal under a weak knock condition at 5200 rpm.

FFT is used to calculate the frequency of the IMF3 component, and the result is shown in Figure 18. From Figure 18, the knock characteristic frequency of the engine block vibration signal under a weak knock condition at 5200 rpm is 3.125 kHz.

## 6. Comparison and Discussions

**Figure 19:**
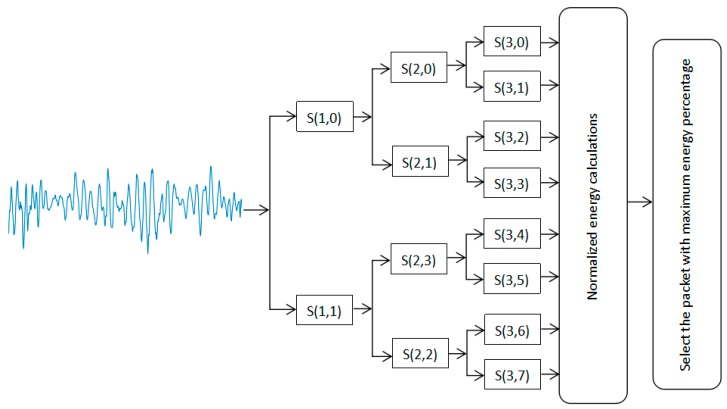
Block diagram of the Wavelet Packet Energy method used to extract the knock characteristics of the engine block vibration signal under a weak knock condition at 5200 rpm.

To verify the effectiveness of the IMFE method, Wavelet Packet Energy method was used to identify the weak knock characteristics from the engine block vibration signal at 5200 rpm, as shown in Figure 15b. A Wavelet Packet Energy method decomposes the engine block vibration signal into a library of orthonormal bases organized in a binary tree structure [40]. Then, the normalized energies of the packets at level 3 are calculated. Figure 19 illustrates the generalized block diagram of the proposed approach.

**Figure 20:**
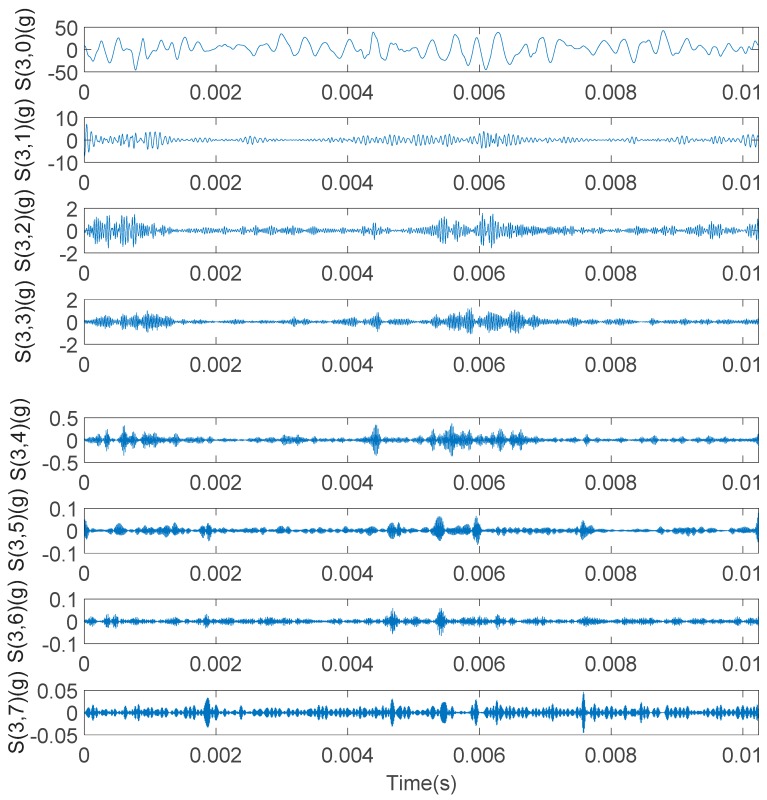
The reconstruction signals corresponding to eight nodes in the third layer.

As can be seen from Figure 19, the third layer of the wavelet tree has eight nodes. Eight nodes in the third layer are reconstructed and sorted, respectively, and the reconstruction signals corresponding to each node are obtained, as shown in Figure 20.

**Figure 21:**
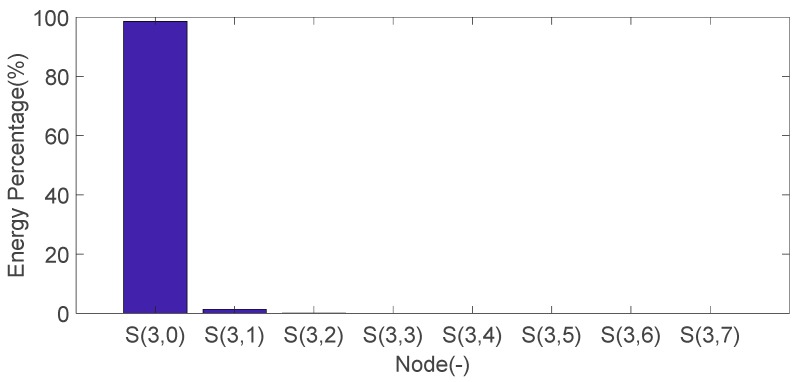
The energy percentage for the reconstructed signal of eight nodes in three layers.

Calculate the energy percentage for the reconstructed signal of eight nodes in three layers, as shown in Figure 20. The calculation result of the energy percentage of the reconstructed signal is shown in Figure 21. It can be seen that the reconstructed signal of node S (3,0) has a maximum energy weight value, and its value is 98.56%.

**Figure 22:**
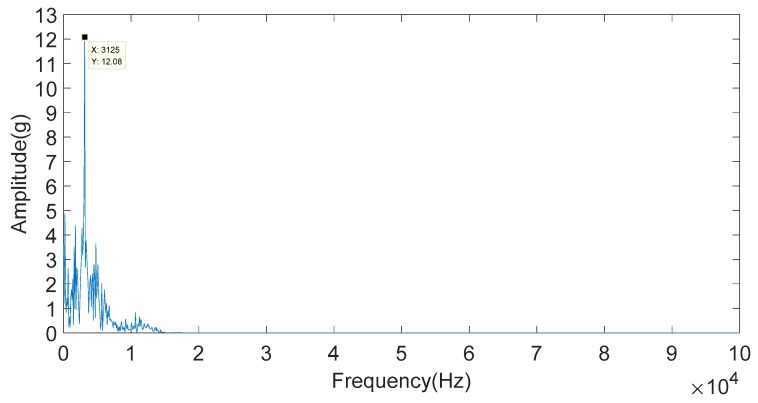
Frequency characteristics of reconstructed signals of node S (3,0).

The reconstructed signal of node S (3,0) was analyzed by FFT, and its frequency characteristics were obtained, as shown in Figure 22. It can be seen that the frequency value is 3.125 kHz. Essentially, the knock frequency of the engine block vibration signal at 5200 rpm, as shown in Figure 15b, is 3.125 kHz. This is consistent with the frequency obtained by the IMFE method, as shown in Figure 18. Thus, the effectiveness of the IMFE method in extracting the characteristic frequency of engine block vibration signals under a weak knock condition is proven.

## 7. Conclusions

In this paper, the IMFE method is used to extract the knock characteristic frequency of engine block vibration signals of a two-stroke SI UAV engine burning RP-3 kerosene fuel under a weak knock condition. In this way, engine block vibration signals are decomposed into a series of IMF components in the frequency domain adaptively. Calculate the energy weight value of each IMF component, and select the component with the largest energy weight value as the dominant characteristic component. By analyzing the spectral characteristics of the main IMF component, the knock characteristic frequency of the vibration signal can be obtained. The results show that the weak knock characteristic frequencies of the engine block vibration signal at 5100 rpm and 5200 rpm are 3.320 kHz and 3.125 kHz, respectively.

A Wavelet Packet Energy method is used to extract the characteristics of the engine block vibration signal of the two-stroke SI UAV engine at 5200 rpm, and the same results are obtained with the IMFE method. This verifies the effectiveness of the IMFE method in extracting the characteristic frequency of an engine block vibration signal under a weak knock condition. The IMFE method does not need to set a fixed basis function, and the extraction of IMF components in each order is determined by the signal characteristics. It is adaptive and has higher accuracy when compared with wavelet transform. It has a certain application value for extracting a characteristic frequency of the engine block vibration signal under a weak knock condition and provides effective theoretical basis for realizing real-time knock control of the two-stroke SI UAV engine.

## Figures and Tables

**Table 1:** Property comparisons of aviation gasoline and RP-3.

Properties	Aviation Gasoline	RP-3
Composition	C5–C11	C7–C16
Relative molecular mass	115	141
Density (kg/m^3^)	720	780
Kinematic viscosity (mm^2^/s) (20 °C)	0.62	1.26
Heat of combustion (kJ/kg)	44,000	43,300
Latent heat of vaporization (kJ/kg)	340	356
Flash point (°C)	−47	40
Octane number	90	46
Auto-ignition temperature (°C)	438	274

**Table 2:** Test engine specifications.

Items	Value
Type of engine	Two-stroke SI engine
Intake style	Reed valve
Stroke/mm	69
Bore/mm	76
Displace/cm^3^	625
Geometric compression ratio	9.5
Exhaust port opening/°CA ATDC	88
Scavenging port opening/°CA ATDC	122

**Table 3:** Operational conditions of the test.

Items	Value
Inlet temperature	301 K
Ambient pressure	101.3 kPa
Sampling frequency	200 kHz
